# Risk factors for breast cancer among women in Ekurhuleni Metropolitan Municipality, Gauteng province of South Africa, 2017‒2020: a case-control study

**DOI:** 10.3332/ecancer.2023.1593

**Published:** 2023-08-25

**Authors:** Sizeka A Mashele, Thembekile B Zwane, Lazarus Kuonza, Mazvita M Muchengeti, Lactatia Motsuku

**Affiliations:** 1 South African Field Epidemiology Training Program, National Institute for Communicable Diseases, National Health Laboratory Services, 1 Modderfontein Road, Sandringham, Johannesburg 2192, South Africa; 2 National Cancer Registry, National Health Laboratory Services, 1 Modderfontein Road, Sandringham, Johannesburg 2192, South Africa; 3 School of Public Health, University of the Witwatersrand, 27 St Andrews Rd, Parktown, Johannesburg 2193, South Africa; 4 South African DSI-NRF Centre of Excellence in Epidemiological Modelling and Analysis (SACEMA), Stellenbosch University, 19 Jonkershoek Road, Stellenbosch 7600, South Africa

**Keywords:** breast cancer, risk factors, incidence, age-standardised incidence rate, case-control study, odds ratio, women, Ekhuruleni Metropolitan Municipality, South Africa

## Abstract

**Introduction:**

Breast cancer (BC) is the most common cancer among women in South Africa (SA), with an age-standardised incidence rate of 52.6 and an age-standardised mortality rate of 16.0 per 100,000 population. There is a paucity of evidence on the risk factors for BC among women of all races in SA. Given the rising prevalence of BC in SA, literature-based evidence is critical for the appropriate dissemination of preventative measures. This study aimed to identify the risk factors associated with the development of BC among women in Ekhuruleni Metropolitan Municipality.

**Methods:**

An unmatched case-control study was conducted from 1 January 2017 to 31 December 2020 using secondary data extracted from the Ekurhuleni Population-Based Cancer Registry. Unconditional multivariable logistic regression analysis was carried out using the adjusted odds ratio (aOR). The variables race, employment, human immunodeficiency virus (HIV), smoking and alcohol status were included in the multivariable logistic regression model while the model was adjusted for age.

**Results:**

A total of 2,217 cases and 851 controls were enrolled in the study. The mean age (±SD) in years was 55.7 (±15.2). The White population group, being self-employed and being HIV positive was significantly associated with reduced odds of BC development. HIV-positive women were 61% less likely to have BC than women who were HIV-negative (aOR 0.39; 95% confidence interval (CI): 0.27‒0.57). White women were 65% less likely to have BC than women of other races (aOR 0.35; 95% CI: 0.29‒0.43). Self-employed women were 59% less likely to have BC than women who were formally employed (aOR 0.41; 95% CI: 0.18‒0.97). No evidence of association was observed between tobacco smoking and BC as well as alcohol consumption and BC.

**Conclusion:**

There was a 65% reduction in BC risk among White women compared to other races. HIV-positive women demonstrated a 61% lower likelihood of BC while self-employed women showed a 59% reduced risk of developing BC. These findings suggest that being White, self-employed or HIV-positive may provide some protection against BC. However, additional research is needed to validate these results and establish the underlying reasons behind these associations.

## Introduction

Breast cancer (BC) is the most common cancer among women [1–3]. In 2020, the number of new cases diagnosed in women was 2.3 million worldwide, with more than 700,000 new cases reported in low-and middle-income countries [4]. With over 9,000 new cases reported in South Africa (SA) in 2020, BC accounted for 23% of all cancers in women [5–7]. Several risk factors have been associated with the development of BC among women worldwide. However, there is a paucity of studies on BC risk factors in SA, as well as a lack of generalisability, due to small sample sizes and a focus on a single population group [8, 9]. There may be cancer risk factors that are peculiar to a given environment but are rarely seen in high-income countries [10].

The National Cancer Institute defines a cancer risk factor as anything that may increase the chances of developing cancer disease [11]. Some risk factors, such as age, race, sex and family or personal history of cancer, are beyond an individual's control [12]. However, behavioural risk factors such as poor eating habits, physical inactivity, tobacco smoking, excessive alcohol consumption, obesity and use of oral contraceptives among others can be controlled or prevented [1, 13, 14]. Reproductive factors, such as late childbearing, low parity, lack of breastfeeding and prolonged exposure to estrogen due to early menarche, late age at first childbirth and late menopause, are well-documented risk factors for BC [8, 15]. The role of certain factors such as race, occupation and residential status in the development of BC in SA remains controversial [16, 17].

A large proportion of BC cases in SA has been observed among the White population group, this may reflect the role of some specific risk factors in this population group [16, 18]. Race has been used as a proxy for access to cancer screening and diagnosis in SA due to historical inequalities [16]. The epidemiology of Human Immunodeficiency Virus (HIV), obesity as well as alcohol consumption differs by race [19, 20]. A study in SA by Vorobiof *et al* [16] reported a doubled incidence rate of BC among women living in urban areas. The BC burden in urban areas is characterised by Westernised behaviours and lifestyles that favour high BC incidence rates [18]. Comprehensive national data on cancer incidence in SA is critical for studying BC risk factors in women across all demographic categories.

To ensure that women are aware of their BC risk in these settings, information on the risk of factors such as race, occupation and residential status among others is crucial. The Ekurhuleni Population-Based Cancer Registry (EPBCR) provides a unique opportunity to achieve such an objective. It contains routinely collected cancer records among individuals living in the Ekhuruleni Metropolitan Municipality (EMM), Gauteng Province (GP) of SA. The EPBCR was established in 2011 by the National Department of Health through the enaction of regulation number 380 of the National Health Act 61 of 2003, to increase cancer reporting and registration in SA [21]. However, no research studies have been conducted using this database. This case-control research study was conducted to identify the possible risk factors for BC in women living in the EMM using the data from the EPBCR.

## Methods

### Study setting and data sources

The study used secondary data extracted from EPBCR collected between 1 January 2017 and 31 December 2020, to identify the risk factors for BC among women. EPBCR is a population-based active cancer surveillance site of the National Cancer Registry of SA. EPBCR collects information on cancer incidence among the residents of the EMM. EMM is located in the East Rand region of GP in SA. It occupies a 1,975 km^2^ area with small towns, townships and informal settlements [22]. It has a population of 3,894,000 persons, a population density of 1,609 persons/km^2^ and a population growth rate of 1.9% in 2020 [22, 23]. It covers about 6% of the SA population and appropriately represents SA’s multiracial diversity with 79% Blacks, 16% Whites, 3% Coloureds and 2% Asians/Indians [22]. EPBCR’s procedures are described in full elsewhere [21, 24–26]. Briefly, cancer incidence data are actively collected from all identified sources within the catchment area of the EPBCR by surveillance officers [22]. The identified sources of EPBCR include private and government healthcare facilities, laboratories, mortuaries, hospices and referral hospitals of EMM [22]. Cancer incidence data are collected using the Research Electronic Data Capture (REDCap) system hosted in Johannesburg, SA by the University of the Witwatersrand [27, 28]. The cases are coded using the International Classification of Disease for Oncology version 3 guidelines and stored within the REDCap system.

### Study design and population

We conducted an unmatched case-control study to identify risk factors for BC in women between 2017 and 2020. The study included all women who were registered in the EPBCR database with BC diagnosed between 1 January 2017 and 31 December 2020. The study was conducted using a study design methodology described by Sengayi-Muchengeti *et al* [29] in a study that was published in 2021 to investigate the evolving association between HIV and cancer in the context of antiretroviral treatment (ART) availability as well as compare to the available literature on HIV-associated cancers in Africa. The methodology allows for case-control studies for any cancer to use other cancers as controls. A case was defined as any woman residing in EMM with a primary BC diagnosis during the study period. All women with residential addresses outside of the EMM were excluded. BC cases that were recorded as being diagnosed by another mode of BC diagnosis except for clinical, histology, fine needle aspiration (cytology), death certificates, as well as autopsy, were excluded. Controls were defined as any cancers diagnosed among EMM women, except for BC as well as hormonal-, smoking- and alcohol-related cancers during the study period. The selection of controls was conducted using an established case-control methodology for four scenarios for potential control selection for cancer types as described by Chen *et al* [30]. Cancers of the bone, endocrine gland, melanoma, myeloma, small intestine, testis, eye, Hodgkin lymphoma, non-Hodgkin lymphoma, non-myeloid leukaemia and squamous cell were used as controls. Among these controls, none of them are hormonal-, smoking- and alcohol-related cancers and their association is null. All males, non-residents of EMM, women under 18 years old and those with cancer diagnosis before or after the study period, women who were not registered in the EPBCR database during the study period, as well as metastatic and primary site unknown cancers, were excluded.

### Sampling procedure

**Figure 1.**
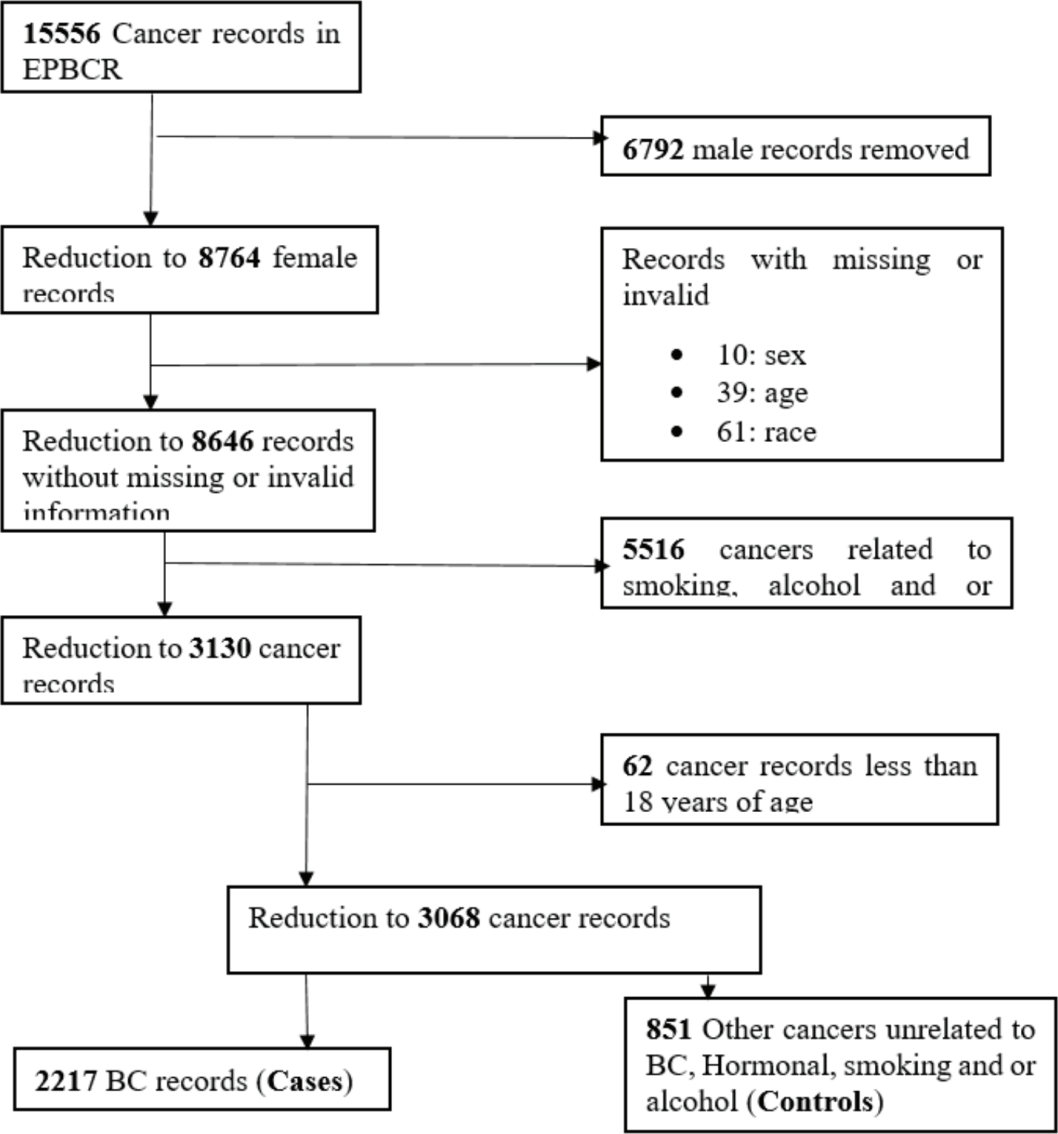
Sampling diagram of women included in the study as cases and controls, 2017‒2020.

All women who met the requirements for case and control definition were enrolled in the study. A total sample of 3,068 (2,217 cases and 851 controls) was extracted from the EPBCR database (Figure 1). Case-control studies with more cases than controls are not common; however, they are practised in certain unique circumstances [31]. Several power calculations were done with the various candidate's risk factors such as race, HIV status, smoking status and alcohol consumption. The candidate's risk factor which could yield the largest power to detect at least 50% odds of BC was the participants with a history of employment. The power of the study was calculated using Stata IC version 15.1 based on a 95% confidence interval (CI), 31.0% of cases and 24.0% of controls with no history of employment. A 97.4% power of the study was achieved.

### Data cleaning and analysis procedure

Before analysis, all data were checked for completeness and internal consistency and then cleaned and analysed using Stata IC version 15.1. Observations with missing values on key variables such as age and sex were dropped from the analysis. Descriptive statistics such as means for normally distributed continuous variables, frequencies and proportions for categorical variables were used to summarise the socio-demographic and clinical characteristics of the participants. A univariate logistic regression analysis was conducted for all the variables and adjusting for age. Every variable was incorporated into the model for the multivariable logistic regression analysis. The variables included in the multivariable logistic regression model were race, employment, HIV, smoking and alcohol status and the model was adjusted for age. A *p*-value of ≤0.05 was considered statistically significant.

## Results

### Socio-demographic characteristics of cancer-diagnosed women

A total sample of 3,138 newly diagnosed cancer patients were enrolled in the study from 2017 to 2020. We excluded 49 participants for missing sex (0.3%) and age (1.9%), 62 participants were less than 18 years (1.9%) of age and 8 (0.2%) participants with *in-situ* cancers for a final sample of 3,068 participants. There was a final total of 2,217 cases and 851 controls. The participants' mean (±SD) age at diagnosis was 55.7 (±15.2) years (cases = 54.8 (13.9) and controls = 58.0 (17.9)), with the age range of 18–105 years old. The majority of the cases (*n* = 1,560) and controls (*n* = 572) were among the age groups 38–67 years. Among all participants enrolled, 1.7% (*n* = 43 cases and *n* = 10 controls) were Asian, 56.6% (*n* = 1,382 cases and *n* = 354 controls) were Black, 1.9% (*n* = 49 cases and *n* = 10 controls) were Coloured and 39.8% (*n* = 743 cases and *n* = 477 controls) were White women. A significantly higher proportion of Black women (*n* = 1,382, 62.3%) had developed BC compared to Asian, Coloured and White women (*p* < 0.001). About 8.1% (*n* = 180) of cases and 6.3% (*n* = 54) of controls were alcohol consumers while 8.2% (*n* = 182) of cases and 6.6% (*n* = 56) of controls were tobacco smokers. Regarding occupational status, about 24% of all participants were unemployed while 8.5% were employed, with <1% of patients who have reported self-employment (Table 1).

### Clinical characteristics of cancer-diagnosed women

Among the total participants, 9.6% (*n* = 206 cases and *n* = 90 controls) were reported to be HIV positive at diagnosis. Among all participants, 386 participants were diagnosed with cancer stage two (stage II) while a majority (*n* = 2,258) of the participants had unknown cancer stage at diagnosis. A larger proportion (*n* = 2,854, 93.0%) of participants had cancer diagnoses confirmed through histological means of cancer diagnosis. A significantly higher proportion of women who were HIV negative (*n* = 352, 11.5%) had developed BC compared to those who were HIV positive (*p* < 0.001) (Table 2).

### Identification of risk factors for BC

In the multivariable logistic regression analysis, race, employment and HIV status were significantly associated with reduced odds of BC development among women. White women were 65% less likely to have BC than Black women adjusted odds ratio (aOR 0.35; 95% CI: 0.29‒0.43). Self-employed women were 59% less likely to have BC than women who were in formal employment (aOR 0.41; 95% CI: 0.18‒0.97). HIV-positive women were 61% less likely to have BC than women who were HIV-negative (aOR 0.39; 95% CI: 0.27‒0.57 CI). We could not identify a statistically significant association between tobacco smoking and BC as well as alcohol consumption and BC development (Table 3).

## Discussion

This study enrolled 3,068 women diagnosed with cancer in EMM over 4 years (2017–2020) to identify risk factors for BC among women. The study found that the White population group, self-employment and being HIV-positive were significantly associated with lower odds of BC development in EMM. There was no evidence of association identified between BC and alcohol consumption as well as BC and tobacco smoking.

Several studies have been conducted to describe the risk factors for BC globally [12, 32–37]. The study by Williams *et al* [33] conducted to understand the social context of BC risks in African American women found a lower risk of BC among White women. BC is a genetic and lifestyle disease. Lifestyle factors such as breastfeeding and obesity are likely to be the factors that are associated with the reduced odds of BC among White women compared to Black women. A study by Jones *et al* [34] shows that Black women in urban areas are likely not to breastfeed or are likely to discontinue breastfeeding as compared to White women. Socioeconomic disparities between Black and White women are among the reason for not breastfeeding or discontinuing breastfeeding in Black women due to the need to go back to work or start a new job [20]. The need for employment has led to many children being introduced to complementary feeding, which anyone can do when the mother is at work. EMM is an urban area in the GP of SA which is an economic hub of SA where people migrate from other provinces to seek employment and a better living. Nglazi and Ataguba [20], also highlighted the high prevalence of obesity among urban Black women in SA as compared to White women. This may also explain the reduced odds of BC among White women in EMM. Another explanation for the high incidence of BC among the Black population in EMM is the occupational exposure to risk factors due to historical reasons where Black people did not share similar occupations with White people, and environmental exposure to risk factors due to differences in living arrangements amongst Black and White people in SA [20].

Self-employment and HIV status also play a role in the reduced odds of BC among White women compared to Black women. Historically, Black communities in SA have faced barriers to accessing quality healthcare due to apartheid-era policies and ongoing socio-economic disparities. Limited access to healthcare services, including cancer screening and treatment facilities, can result in delayed diagnoses and poorer outcomes for Black women [38]. In this study, there is a higher proportion of self-employed women among White women as compared to Black women. SA has one of the highest HIV prevalence rates globally, and HIV disproportionately affects Black women in SA. The prevalence of HIV is higher among Black women compared to White women [39]. HIV can complicate the diagnosis and treatment of BC in several ways such as HIV-related immunosuppressions, which might lead to delays in the initiation of cancer diagnosis. Considering the high HIV prevalence in SA, this may have implications for BC incidence among HIV-positive women in the country. This is likely one of the reasons for the lower odds of BC among White women in EMM. Singh *et al* [40] previously found that White women in SA have a higher incidence of BC. This study found a higher incidence of BC among Black women. The contradiction may also be likely due to the sources of BC data in the EPBCR. Investigation into the sources of BC data is likely to clarify the contradictions outlined.

We have identified that self-employed women have lower odds of BC as compared to women who are employed. Several industries have been associated with an increased risk of BC [19, 41]. Some agricultural employment such as those that use pesticides are associated with the risk of BC development [42]. Most of the self-employed women are likely to have employed others, they are also likely not to involve themselves frequently on the ground or in baseline activities. Self-employed women often have more control over their work schedule, which may enable them to make healthier lifestyle choices. They are likely to have more time for regular physical activity, better meal planning and reduced exposure to workplace stressors. This may somehow explain the lower odds of BC among women in this setting. Being self-employed could allow women to have more flexibility to seek healthcare services such as scheduling medical check-ups and screenings, including mammograms. Although women who are not self-employed might find it more difficult to take a day off from work to go to healthcare facilities if they want a check-up or they are ill, regular screening can aid in early detection and improve treatment outcomes. Self-employed women are also likely to have different socioeconomic backgrounds and access to healthcare compared to employed women. These factors can influence BC risk.

HIV-positive women had lower odds of BC as compared to women who were HIV-negative. There is a paucity of evidence on the lower odds of BC in HIV-positive women, however, the study conducted in the United States of America to determine the incidence of BC among people living with HIV (PLWH) found a lower incidence rate among the people living with HIV/AIDS (PLWHA) as compared to the HIV-negative population [12]. Another study by D’Andrea *et al* [32] to identify the possible links existing between HIV infection, highly active antiretroviral therapy and BC risk found that PLWHA has slightly lower odds of BC when compared to the general population.

Like any other cancer type, BC is an age-associated disease. In this study, about 82% of cases were diagnosed with BC at the age of 40 years old and above. In SA, the prevalence of HIV is higher (19.8%) in younger women aged between 15 and 39 years old [12, 29]. Because of the fact highlighted above, we have a small proportion (9.3%) of HIV-positive cases registered in the EPBCR. The risk of BC increases with age, however, the HIV population is primarily young in SA. The reduced odds of BC among HIV-positive women might also be explained by the ART rollout in SA. The studies by Williams *et al* [33] and D’Andrea *et al* [32] highlight the importance of ART in BC epidemiology. We do not have evidence of HIV-positive BC cases being enrolled in the ART program in this study. However, in SA, before 2004, mortality due to HIV was high with a low survival rate and reduced life expectancy [33]. After the implementation of the ART program, low mortality due to HIV and improved life expectancy were observed among PLWHA [33, 43].

When we investigated alcohol consumption and tobacco smoking on the risk of BC, we could not establish a significant association with BC in this population. Studies demonstrated that alcohol consumption and smoking increase BC risk in a dose-dependent manner [44, 45]. Active and passive variations, as well as personal behaviour in tobacco smoking, have also been cited in the association between smoking and BC [46]. A large proportion of incompleteness of smoking and alcohol variables may likely explain the no association between these variables and BC.

Our study had several limitations. The fact that we have used secondary data rather than primary data limited us to exploring only factors that were available in the dataset. Therefore, other factors of importance such as occupational factors, reproductive factors as well as details on smoking and drinking behaviours could not be explored. Also, this study used controls that are diagnosed with different cancer types. While we acknowledge that this type of control selection may introduce unexpected referral bias, careful selection of controls was done using an established cancer control selection method [30]. As outlined in previous studies [29, 30], a careful selection of controls produces prevalence rates that resemble background levels. The large proportion (>60%) of missing information observed on the variables such as alcohol consumption, tobacco smoking, HIV and employment status may have introduced some levels of bias in the analyses and therefore need for further studies in this regard. We have also noted that using residential addresses alone could create a bias of including participants that are not permanent residents of EMM. This is one of the limitations of this data because it is impossible to validate how long the participants stayed in a particular address since this information is also not available in the hospital patient records/files.

Our study also had some strengths. This is the first study to explore the factors associated with the risk of BC using multiracial population-based cancer registry data in SA. As previously outlined in previous studies [13, 21, 25, 30, 40], this setting mimics the structure and cultural background of SA, therefore, the representativeness was adequate. Our study found lower odds of BC in the White population group, lower odds of BC among HIV-positive women and lower odds of BC among self-employed women. There were no identified risk factors associated with the development of BC in our study. Further analyses of lifestyle, reproductive and environmental factors may provide further insight into the determinants of BC in this setting. The protective effects of HIV and the White population group need confirmation in this setting. It is now pertinent to conduct a primary study to determine the risk factors for BC in EMM. Primary studies will allow for the inclusion of other important factors such as reproductive and behavioural factors among others. There is also a need to improve data collection tools to allow for the collection of other variables of importance in this cancer registry.

## Conclusion

According to the findings, there was a 65% lower likelihood of BC among White women. In addition to White women, HIV-positive women were 61% less likely, while self-employed women were 59% less likely to have BC. These results suggest that being White, self-employed or HIV-positive may act as a protective factor against BC. However, further research is necessary to validate these findings and better understand the reasons behind these outcomes.

## List of abbreviations

aOR, adjusted odds ratio; ART, antiretroviral treatment; CI, confidence interval; EMM, Ekurhuleni Metropolitan Municipality; EPBCR, Ekurhuleni Population-Based Cancer Registry; GP, Gauteng Province; NCR, National Cancer Registry; PLWH, people living with HIV; PLWHA, people living with HIV/AIDS; REDCap, Research Electronic Data Capture; SAFETP, South African Field Epidemiology Training Programme; WHO, World Health Organisation.

## Conflicts of interest

No competing interests are declared by the authors.

## Funding

This study was equally supported financially by the NCR and SAFETP. The publication fees were covered by the University of the Witwatersrand, Johannesburg.

## Ethical considerations

This study was ethically cleared by the Human Research Ethics Committee (HREC) for health science-related research at the University of the Witwatersrand, Johannesburg, SA (Ref. No. M220374). Following approval, written data access permission was obtained from the NCR. Confidentiality of information was maintained.

## Data sharing statement

All data for this study is available upon reasonable request from the corresponding author.

## Author contributions

Sizeka Mashele, Lactatia Motsuku and Mazvita Muchengeti conceived the study. Sizeka Mashele conducted data analysis and interpretation of the results and wrote the manuscript. Lactatia Motsuku provided the data and interpretation of the results and gave critical comments. Thembekile Zwane, Lazarus Kuonza and Mazvita Muchengeti gave critical comments on the manuscript. All authors approved the final draft of the manuscript.

## Figures and Tables

**Table 1.** Socio-demographic characteristics of women diagnosed with cancer in EMM, SA, 2017‒2020.

Characteristics	Controls (*n* (%)) *N* = 851	Cases (*n* (%)) *N* = 2,217	*p*-value
Age cohort			
18–27	45 (5.3)	27 (1.2)	<0.001
28–37	95 (11.2)	203 (9.2)	
38–47	113 (13.3)	509 (23.0)	
48–57	151 (17.7)	524 (23.6)	
58–67	154 (18.1)	527 (23.8)	
68–77	154 (18.1)	307 (13.8)	
78+	139 (16.3)	120 (5.4)	
Race			
Asian	10 (1.2)	43 (1.9)	<0.001
Black	354 (41.6)	1,382 (62.3)	
Coloured	10 (1.2)	49 (2.2)	
White	477 (56.1)	743 (33.5)	
Alcohol consumption			
No	113 (13.3)	503 (22.7)	<0.001
Yes	54 (6.3)	180 (8.1)	
Undisclosed	684 (80.4)	1,534 (69.2)	
Tobacco smoking			
No	131 (15.4)	565 (25.5)	<0.001
Yes	56 (6.6)	182 (8.2)	
Undisclosed	664 (78.0)	1,470 (66.3)	
Employment status			
Employed	74 (8.7)	188 (8.5)	<0.001
Self-employed	13 (1.5)	13 (0.6)	
Unemployed	264 (31.0)	531 (24.0)	
Undisclosed	500 (58.8)	1,485 (67.0)	

**Table 2.** Clinical characteristics of women diagnosed with cancer in EMM, SA, 2017‒2020.

Characteristics	Controls (*n* (%)) *N* = 851	Cases (*n* (%)) *N* = 2,217	*p*-value
HIV status			
Negative	70 (8.2)	352 (15.9)	<0.001
Positive	90 (10.6)	206 (9.3)	
Undisclosed	691 (81.2)	1,659 (74.8)	
Cancer stage at diagnosis			
Stage I	59 (6.9)	118 (5.3)	<0.001
Stage II	57 (6.7)	329 (14.8)	
Stage III	34 (4.0)	165 (7.4)	
Stage IV	17 (2.0)	31 (1.4)	
Unknown	684 (80.4)	1,574 (71.0)	
Basis of diagnosis			
Autopsy	2 (0.2)	1 (<1)	<0.001
Clinical	55 (6.5)	62 (2.8)	
Cytology (Fine needle aspiration)	22 (2.6)	45 (2.0)	
Death certificate only	2 (0.2)	11 (0.5)	
Histology	762 (89.5)	2,092 (94.4)	
Unknown	8 (0.9)	6 (0.3)	

**Table 3.** Factors associated with BC among women in EMM, SA, 2017–2020.

Characteristics	Controls (*n* (%))	Cases (*n* (%))	Total (*n* (%))	OR (95% CI)	*p*-value	aOR (95% CI)	*p*-value
Race							
Black	354 (41.6)	1,382 (62.3)	1,736 (56.6)	1.00		1.00	
Asian	10 (1.2)	43 (1.9)	53 (1.7)	1.11 (0.55‒2.23)	0.767	0.99 (0.49‒2.00)	0.974
Coloured	10 (1.2)	49 (2.2)	59 (1.9)	1.26 (0.63‒2.52)	0.509	0.96 (0.49‒2.01)	0.991
White	477 (56.1)	743 (33.5)	1,220 (39.8)	0.41 (0.35‒0.49)	**<0.001**	0.35 (0.29‒0.43)	**<0.001**
Alcohol status							
No	113 (13.3)	503 (22.7)	616 (20.1)	1.00		1.00	
Yes	54 (6.3)	180 (8.1)	234 (7.6)	0.77 (0.53‒1.11)	0.162	1.22 (0.80‒1.85)	0.347
Smoking status							
No	131 (15.4)	565 (25.5)	696 (22.7)	1.00		1.00	
Yes	56 (6.6)	182 (8.2)	238 ( 7.8)	0.78 (0.54‒1.11)	0.166	1.09 (0.73‒1.63)	0.676
Employment status							
Employed	74 (8.7)	188 (8.5)	262 (8.5)	1.00		1.00	
Self employed	13 (1.5)	13 (0.6)	26 (0.8)	0.38 (0.17‒0.85)	**0.019**	0.41 (0.18‒0.97)	**0.042**
Unemployed	264 (31.0)	531 (24.0)	795 (25.9)	0.85 (0.85‒1.52)	0.306	0.91 (0.65‒1.28)	0.601
HIV status							
Negative	70 (8.2)	352 (15.9)	422 (13.8)	1.00		1.00	
Positive	90 (10.6)	206 (9.3)	296 (9.6)	0.41 (0.29‒0.57)	**<0.001**	0.39 (0.27‒0.57)	**<0.001**

OR: odds ratio; aOR: adjusted odds ratio

Bold: statistically significant associations at *p* ≤ 0.05
